# Real world performance of cardiac magnetic resonance imaging in patients with clinical indications: initial experience from a tertiary referral hospital in China

**DOI:** 10.1186/1532-429X-16-S1-P126

**Published:** 2014-01-16

**Authors:** Yucheng Chen, JIayu Sun, Xin Liu

**Affiliations:** 1 Department of Cardiology, West China Hospital, Chengdu, China; 2 Department of Radiology, West China Hospital, Chengdu, China; 3 Paul C. Lauterbur Research Center for Biomedical Imaging, Shenzhen, China

## Background

Cardiac magnetic resonance (CMR) has been developed as an important imaging tool for cardiovascular diseases in clinical practice. However, there were few reports about the real world implementation of CMR with regard to its indications and findings.

## Methods

344 patients (48 ± 19 yrs, 63.4% male) with clinical indications for CMR were consecutively recruited in 10 months, at a tertiary referral hospital in China. Indications for CMR, quantifications of structure and function, late gadolinium enhancement (LGE), and diagnoses made by CMR were analyzed.

## Results

The evaluation of heart failure and cardiomyopathies was the leading cause of referral that occurred in 164 (47.7%) patients, followed by coronary artery disease in 79 (23.0%) patients and ventricular arrhythmia in 65 (18.9%) patients. Quantitative analysis was available in 319 (92.7%) patients where the LV dilatation observed in 208 (65.2%) patients and RV dilatation in 46 (14.4%) patients, respectively. There were 157 (48.5%) patients detected with ventricular LGE, in whom 43 (27.4%) patients displayed typical ischemic pattern and the others had non-ischemic pattern of various presentations. Except the undefined 74 (21.5%) cases, CMR assessment corrected an initial referral diagnosis in 73 (21.2%) patients and confirmed it in 197 (57.3%) patients.

## Conclusions

All the referrals in this study were appropriate for CMR, mainly indicated for structure and function assessment. CMR helped to identify abnormalities that not detected by other imaging modalities, which would provide incremental diagnostic, therapeutic and prognostic information.

## Funding

This study was supported by a grant from the National Science Funds of China (81271531).

